# Supplementary material for: A reproducible and generalizable software workflow for analysis of large-scale neuroimaging data collections using BIDS Apps
Source: Imaging Neurosci (Camb). 2024 Jan 25;2:imag-2-00074. doi: 10.1162/imag_a_00074 (PMC12224434; doi:10.1162/imag_a_00074)
Supplement: Supplementary Material [file imag_a_00074-supp.pdf]

SUPPLEMENT

NMIND checklist

The NMIND checklist is a tool to evaluate the scientific software against coding standards proposed by NMIND consortium (Kiar et al., 2023). The full checklist can be found here: https://www.nmind.org/standards-checklist/. Here we show our Python package BABS's performance on this checklist (checklist version: 1.0.0; evaluated on October 30th, 2023). This checklist includes three domains: documentation, infrastructure, and testing. The assessment for each domain could land on one of these three tiers: Bronze, Silver, Gold. The achieved items are labeled with checkmarks "√", while not achieved ones are labeled with open circles "○".

It should be noted that, current checklist is based on the latest version of BABS when the paper is submitted. As BABS is under continued development, after more features are added, more checks may be achieved than those listed below, and BABS may even reach a higher tier.

Domain #1: Documentation

Bronze tier (9 out of 9 items have been achieved):

√ Landing page (e.g., GitHub README, website) provides a link to documentation and brief description of what program does

√ Documentation is up to date with version of software

√ Typical intended usage is described

√ An example of its usage is shown

√ Document functions intended to be used by users (i.e., public function docstring / help coverage ≥ 10%)

√ Description of required input parameters for user-facing functions with reasonable description of inputs (i.e., "NIfTI of brain mask in MNI" vs. "An image file")

√ Description of output(s)

- √ User installation instructions available
- √ Dependencies listed (i.e., external and within-language requirements)

Silver tier (7 out of 7 items have been achieved):

- √ All items from bronze tier
- √ Background/significance of program
- √ One or more tutorial to showcase the multiple of the program's usages (i.e., if program has multiple usages)
- √ Any alternative usage that is advertised is thoroughly documented
- √ Thorough description of required and optional input parameters
- √ Document public functions (i.e., public function docstring / help coverage ≥ 20%)
- √ A statement of supported operating systems / environments (i.e., could be a container recipe)

Gold tier (4 out of 8 items have been achieved):

- √ All items from bronze tier
- √ All items from silver tier
- o Continuous integration badges in README for build status
- √ Continuous integration badges in README for tests passing
- o Continuous integration badges in README for coverage
- √ Document functions, classes, modules, etc. (i.e., public + private docstring / help coverage ≥ 40%)
- o Has a documented style guide
- o Maintenance status is documented (e.g., expected turnaround time on pull requests, whether project is maintained)

Domain #2: Infrastructure

Bronze tier (7 out of 7 items have been achieved):

- √ Code is open source
- √ Package is under version control
- √ Readme is present

√ License is present
√ Issues tracking is enabled (i.e., either through GitHub or external site)
√ Digital Object Identifier (DOI) points to latest version (e.g., Zenodo)
√ All documented installation instructions can be successfully followed

Silver tier (4 out of 4 items have been achieved):

√ All items from bronze tier
√ Issue template(s) available (i.e., information requested by developers)
√ Continuous integration runs tests
√ No excessive files included (i.e., unused files / cache; e.g., .gitignore)

Gold tier (4 out of 7 items have been achieved):

√ All items from bronze tier
√ All items from silver tier
√ Continuous integration builds packages
o Continuous integration validates style
o Journal of Open Source Software submission
√ Contribution guide present
o Code of Conduct present

Domain #3: Testing

Bronze tier (2 out of 2 items have been achieved):

√ Provide / generate / point to test data
√ Provide instructions for users to run tests that include instructions for evaluation for correct behavior

Silver tier (2 out of 3 items achieved):

√ All items from bronze tier
√ Some form of testing suite present
o Test coverage > 50%

Gold tier (2 out of 4 items have been achieved):

- √ All items from bronze tier
- o All items from silver tier
- o Test coverage > 90%
- √ Benchmarking information is provided for examples
